# Multiple ossified intracranial and spinal meningiomas: a rare case report and literature review

**DOI:** 10.3389/fneur.2023.1253915

**Published:** 2023-10-11

**Authors:** Jian Wang, Anbang Zhang, Boya Wang, Jingmeng Yuan, Junchi Zhu, Mengjiao Li, Henli Liu, Lijuan Cheng, Ping Kong

**Affiliations:** ^1^Department of Neurology, Affiliated Aerospace Hospital of Zunyi Medical University, Zunyi, Guizhou, China; ^2^Department of Neurology, The First Affiliated Hospital of Guizhou University of Traditional Chinese Medicine, Guiyang, Guizhou, China; ^3^Department of Neurology, People's Hospital of Fenggang County, Zunyi, Guizhou, China

**Keywords:** ossified spinal meningiomas, ossified intracranial meningiomas, multiple, meningiomatosis, case report

## Abstract

Ossified intracranial meningiomas (OIM) and ossified spinal meningiomas (OSM) are rare neoplasms of mesenchymal origin that predominantly manifest in the spinal cord and infrequently in the cranial region, accounting for ~0. 7–5.5% of all meningiomas. It is extremely rare to have multiple intracranial and spinal lesions accompanied by ossification. Herein, we report this rare case for the first time. A 34-year-old woman presented with paresthesia and limb weakness in the right lower limb and gradually worsened. Approximately half a year later, she could only walk with crutches. Magnetic resonance imaging of the brain and spinal cord showed multiple meningiomas, and histopathological examination confirmed multiple OIM and OSM (WHO grade 1). Multiple OIM and OSM are extremely rare with diverse imaging features, and it is easily confused with other tumors. Histopathological examination is the final diagnostic method.

## Background

Multiple intracranial and spinal meningiomatosis refer to the simultaneous or sequential occurrence of meningiomas in two or more locations, approximately accounting for 1–10% of all meningiomas (1). Metaplastic meningiomas are a subtype of meningiomas characterized by focal mesenchymal differentiation with osseous, cartilaginous, lipomatous, myxoid, or xanthomatous elements (2). Ossified intracranial meningiomas (OIM) and ossified spinal meningiomas (OSM) are rare subtypes of metaplastic meningiomas characterized by diverse clinical symptoms and slow growth. Its imaging manifestations are easily confused with other tumors. There are only dozens of OIM and OSM case reports worldwide, whereas multiple intracranial and spinal cord lesions accompanied by ossification are extremely rare (3), and no relevant literature has been found. Herein, we have reported the first case of concurrent occurrence of multiple OSM and OIM, along with a comprehensive review of the literature. The case reports were conducted strictly in accordance with the CARE guidelines (4).

## Case report

We present a case of a 34-year-old woman who complained of paresthesia in the right lower extremity with limb weakness. She described that, initially, she felt paresthesia in her right lower limb, manifested as abnormal sensation and decreased tactile perception on the right plantar. After 1 month, there was no sensation on the right plantar landing, accompanied by numbness and weakness of the right lower limb and ataxia. After half a year, she had to grasp the bed rail with both hands or get support from family members to get up, and she felt weak and unable to support his waist and back while bending over to tie shoelaces. In addition, she had to make repeated attempts to put her right foot into the shoe and had obvious dragging when walking. The patient had been admitted to external hospitals and was diagnosed with lumbar spine disease.

**Figure 1:**
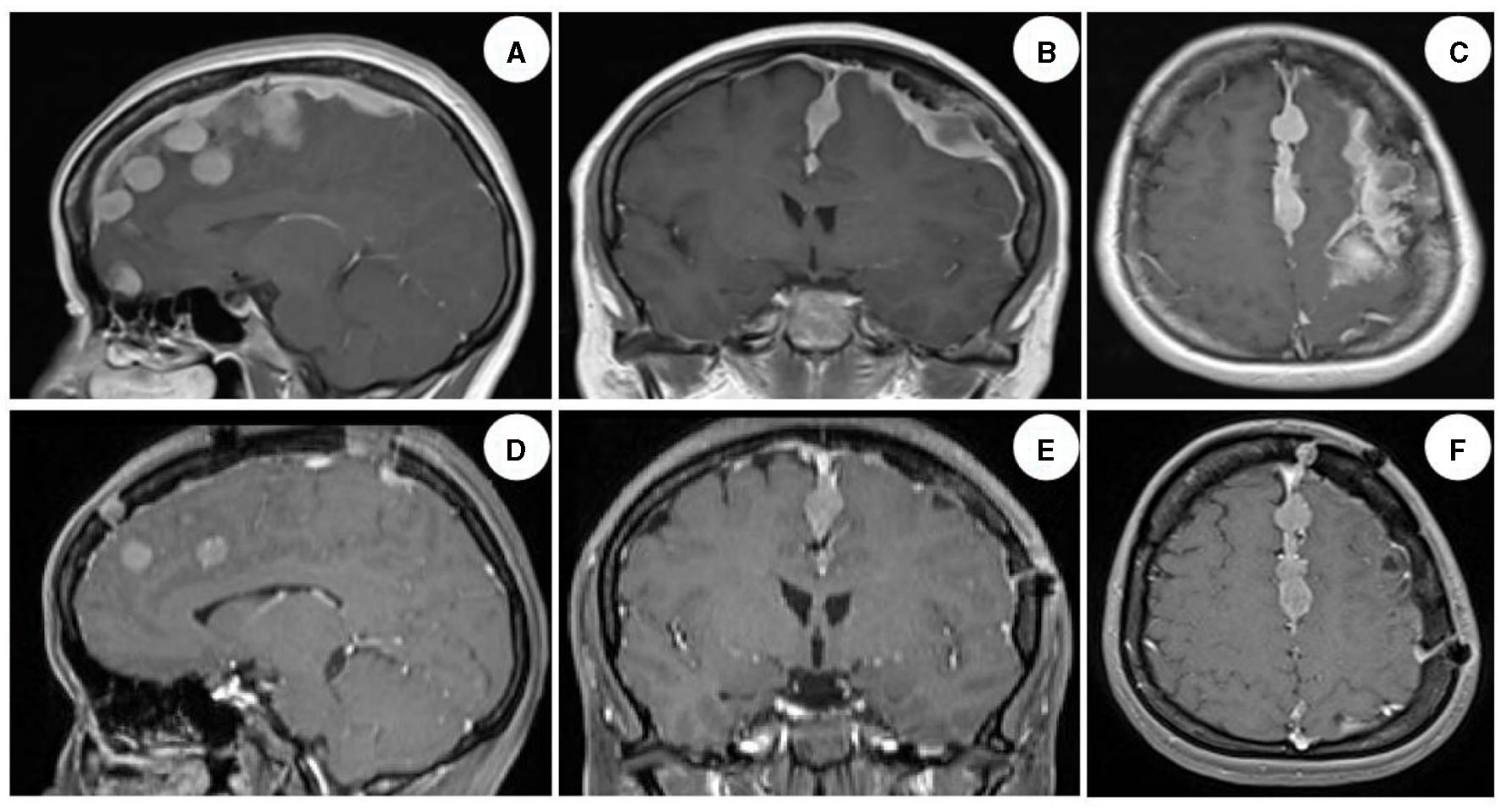
Brain MRI images of the patient. Enhanced images in sagittal, coronal, and transverse views of the brain demonstrated multiple round-like and irregular uniformly enhanced lesions in the left frontoparietal lobe and cerebral longitudinal fissure, with compression and deformation of the brain tissue, deviation of the brain midline, and dural adhesion, accompanied by obvious bone destruction, and no obvious edema around the lesions [**(A–C)** illustrate the given information]. The majority of meningiomas exhibiting evident space-occupying effects were successfully excised, leading to a significant alleviation of brain tissue compression. The restoration of brain midline symmetry was essentially achieved; however, a complete resection of the tumor along the intracranial midline was not accomplished. This observation was confirmed by postoperative imaging data obtained after 6 months [**(D–F)** illustrate the given information].

**Figure 2:**
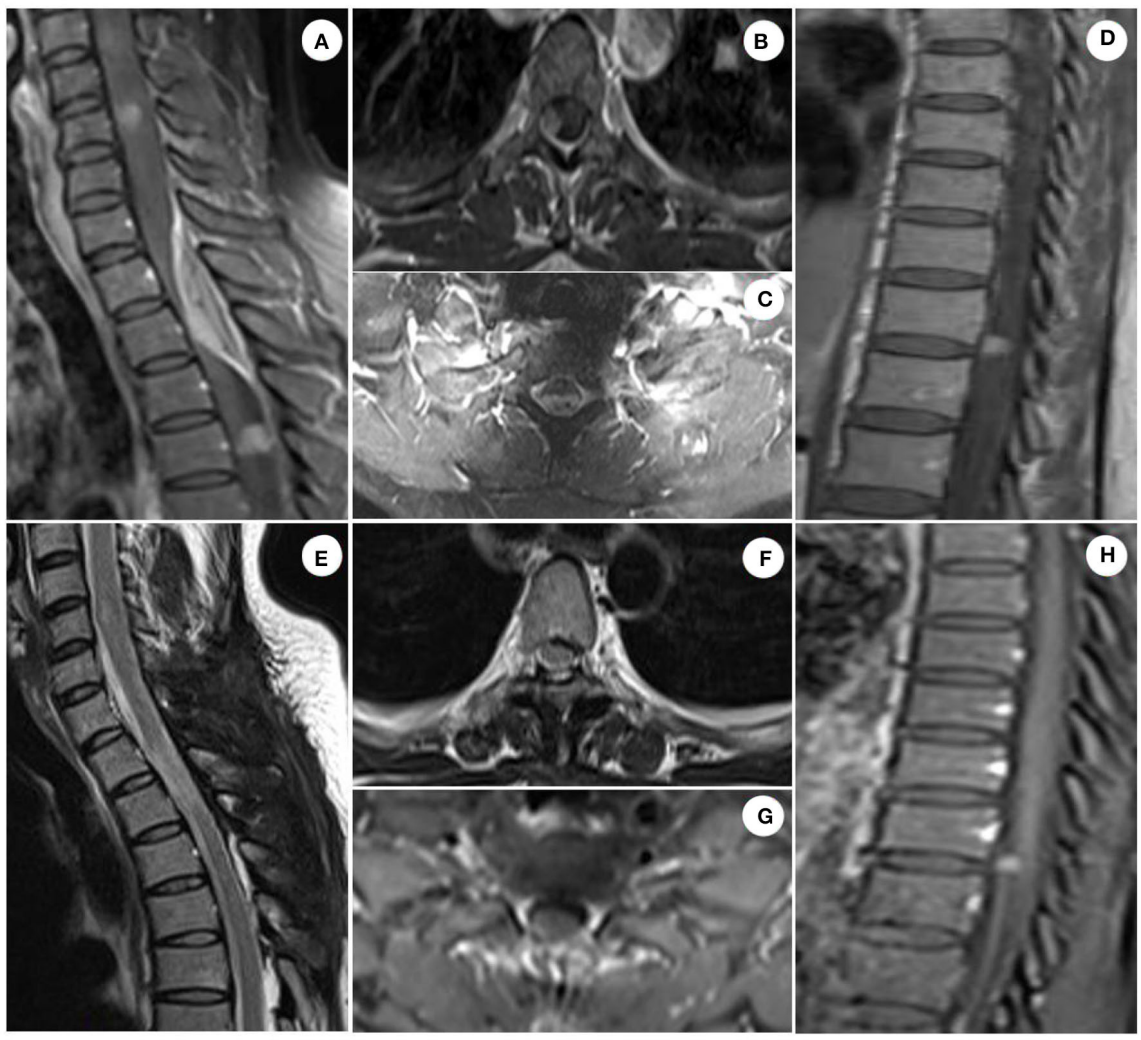
Spinal cord MRI images of patient. Sagittal enhanced T1 scan of the cervical and thoracic spinal cord showed multiple circular and irregular homogeneous enhanced lesions in the spinal cord, and spindle-shaped homogeneous enhanced lesions in the epidural space, with obvious compression and deformation of the spinal cord, accompanied by bone destruction. Axial enhanced T1 scan of the spinal cord demonstrated homogeneous enhancement of the lesion, lower signal intensity in the center of the tumor than that in the periphery, compression, and deformation of the spinal cord, and spinal dural adhesion [**(A–D)** illustrate the given information]. The cervicothoracic spinal cord meningiomas with evident space-occupying effect were successfully excised, leading to significant alleviation of compression on the spinal cord tissue. However, a complete resection of the tumors in the upper cervical and lower thoracic spinal cord was not achievable. This observation was confirmed by postoperative imaging data obtained 6 months later [**(E–H)** illustrate the given information].

**Figure 3:**
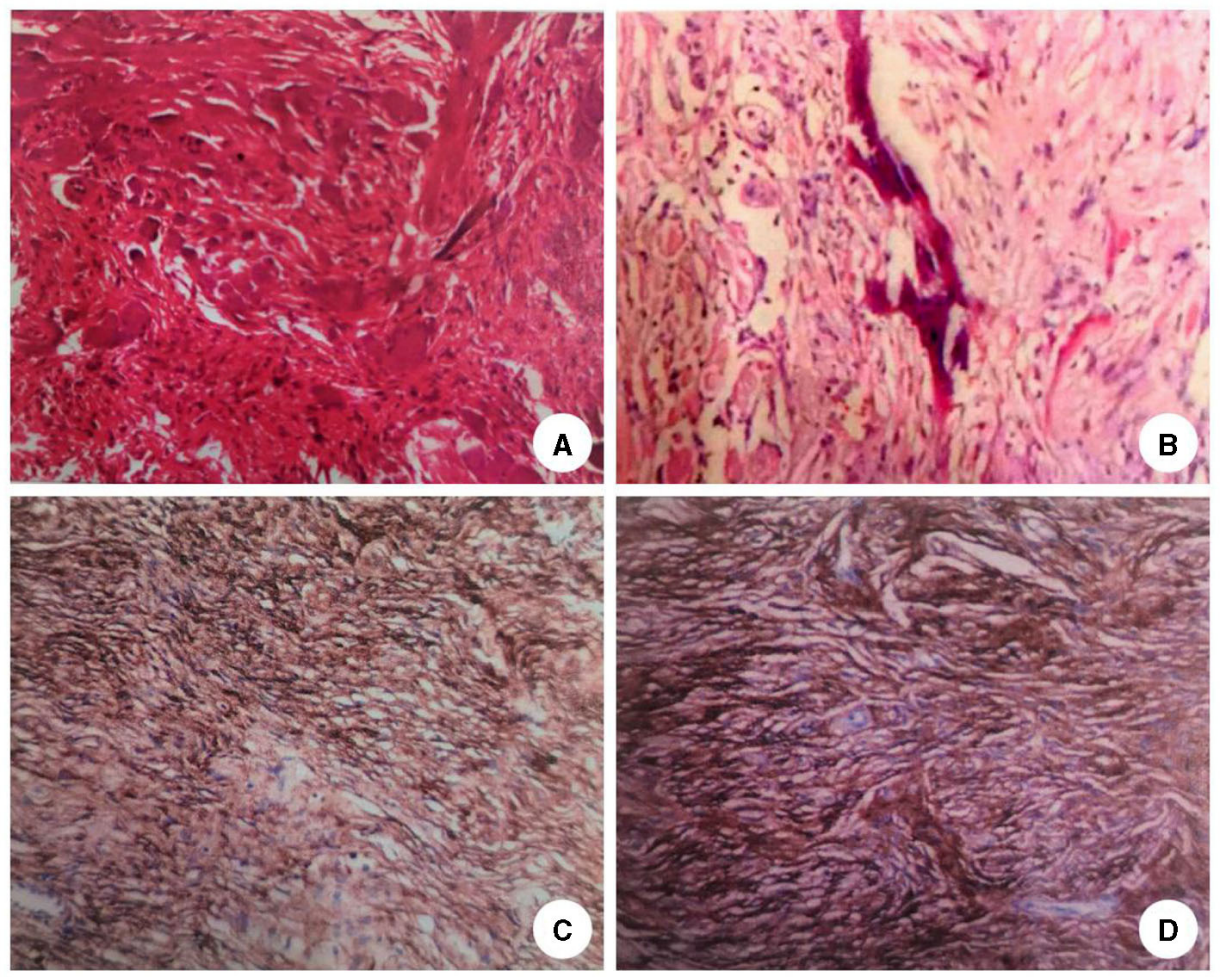
Histopathological results of the lesions. HE staining of the lesion tissue in the left parietal lobe **(A)** and cervicothoracic spinal cord **(B)** showed psammoma bodies around the tumor cells and mature bone tissue formation around the tumor cells (H&E stain, original magnification × 40). Immunohistochemical results of the lesions in the left parietal lobe **(C)** and cervicothoracic spinal cord **(D)** showed that the neoplastic meningothelial cells are immunoreactive for epithelial membrane antigen (EMA) (×10), progesterone receptor (PR) (×10), somatostatin receptor 2 (SSTR2)(×10), and Ki67 ~5%.

The patient was admitted to our department 73 days later due to the progressive deterioration of symptoms that significantly impacted their activities of daily living. Nervous system physical examination showed that the right lower limb proximal muscle strength was grade IV and the distal muscle strength was grade III, the heel knee tibial test of the right lower limb was unstable, and the deep and shallow sensation of the right lower limb was decreased. In addition, Romberg and Babinski signs were positive, the left biceps and triceps brachii demonstrated active reflex, the bilateral knee and ankle showed hyperactivity, and the bilateral patellar and ankle clonus were positive. Moreover, brain magnetic resonance imaging (MRI) showed multiple round lesions in the left frontoparietal lobe and cerebral longitudinal fissure, accompanied by bone destruction, and the lesion was uniformly enhanced during enhancement (Figure 1). The MRI of the spinal cord showed multiple round and irregular lesions in the cervical and thoracic spinal cord with bone destruction, uniformly enhanced during enhancement (Figure 2). Considering the intricate nature and inherent perils associated with surgical intervention, the patient and his family have expressed their desire for a transfer to an alternative medical facility. According to the patient's recollection, he underwent a partial resection of a meningioma at another hospital, encompassing both intracranial and cervicothoracic spinal regions. The postoperative imaging revealed partial excision of the intracranial meningiomas, resulting in significant alleviation of brain tissue compression and remarkable improvement in cervicothoracic spinal cord compression (Figures 1, 2 illustrate the given information). Histopathological examination of the lesion in the left parietal and cervicothoracic spinal cord suggested OIM and OSM (WHO grade 1). Immunohistochemical examination of the lesion showed the EMA (+), PR (+), CD34 (–), GFAP (–), SSTR2 (+), S100 (+), and Ki67 (~3%) (Figure 3). According to the patient's account, paresthesia and weakness in their right lower limb were entirely alleviated through an intensive 3-month rehabilitation program subsequent to their release.

## Discussion

Meningioma is a type of primary central nervous system tumor originating from arachnoid cap cells, accounting for ~25–45% of intracranial tumors, with an incidence rate of 4.7–7.5/100,000 and male/female ratio of 1:2–3.5. In total, 80% of them are benign, sporadic, and solitary (1, 5). Multiple meningiomas, first described by Anfimov and Blumenau in 1889 (6), were defined as the presence of two or more unconnected tumors in the intracranial and extracranial areas without other causes by Cushing and Eisenhardt in 1938, accounting for ~1–10% of all meningiomas. Most of the multiple meningiomas are located in the cranial cavity, whereas few are in the spinal cord (1, 5).

Most meningiomas are sporadic, and familial cases of meningiomas are rare (7). The most common genetic alteration observed in sporadic meningiomas is the deletion of chromosome 22 either in its entirety or distally (7–9). Tumor susceptibility to sporadic meningiomas often arises from heterozygous mutations occurring in the SMARCE1 gene located on chromosome 17q21 (10, 11). Furthermore, heterozygous mutations in SUFU gene (12, 13) on chromosome 10q24 and PDGFB gene (14, 15) on chromosome 22q have also been reported to be associated with the development of meningiomas.

Metaplastic meningioma is a kind of benign tumor originating from arachnoid epithelial cells, which can differentiate into mesenchymal tissues including the bone, cartilage, smooth muscle, and adipose tissues alone or in combination. Most of them grow slowly with a pathological grade of WHO 1, and their clinical symptoms mainly depend on the location of the tumor (16, 17). There is a clear difference between meningiomas ossification and calcification. Calcification is more of an imaging description than a histopathological diagnosis, whereas ossification is a subtype of metaplastic meningiomas characterized by a histopathologic expression of mesenchymal components (18, 19).

OIM and OSM are classified as an uncommon subtype of metaplastic meningiomas, accounting for ~0.7–5.5% of all spinal meningiomas (20). Currently, the mechanisms of OIM and OSM are far from clear. A hypothesis indicated that ossification is caused by the repeated accumulation of hydroxyapatite crystals in the psammoma bodies (21), which has been negated by some reports (22). Most researchers prefer to believe that ossification is secondary to the metaplasia of arachnoid and interstitial cells, which induces a synergistic effect of osteoblasts, fibroblasts, and angiogenic components in bone tissue formation (23–26). Therefore, the theory of mesenchymal differentiation of metaplastic meningioma cells has been proposed (3, 27). The association of the extra-axial mass with dural and osseous reactions, as well as a massive calcified component, may suggest an ossified meningioma. The differential diagnosis may include benign bone processes, such as osteoid osteomas, aneurysmal bone cysts, and fibrous dysplasia, and malignant processes, such as osteogenic sarcomas, chondrosarcoma, and metastatic disease (28, 29).

The expression of epithelial membrane antigen (EMA) and soluble protein-100(S-100) can vary among different meningiomas, which is a well-known phenomenon. However, due to their limited sensitivity and specificity, the combined use of EMA and S-100 is often employed to enhance diagnostic accuracy (30). Progesterone receptor (PR) (31, 32) serves as a highly specific marker for meningiomas. It exhibits high expression in benign meningiomas and low expression in malignant ones. Somatostatin receptor 2 (SSTR2) (33, 34) is currently regarded as the most specific and sensitive biomarker for meningiomas. SSTR2 can be detected in all grades of meningiomas, with high expression observed in benign cases and low expression observed in malignant cases. Studies have shown (34) that the monoclonal antibody for SSTR2a is a highly sensitive and specific marker for meningiomas. SSTR2a is expressed in cases that do not express EMA or PR and that are often considered in the differential diagnosis of meningiomas, including schwannomas, cellular schwannomas, malignant peripheral nerve sheath tumors, and hemangiopericytomas/solitary fibrous tumors. Thus, SSTR2a immunohistochemistry can be useful in establishing the diagnosis of meningiomas, including high-grade meningiomas with poor differentiation. Additionally, Ki-67 (35) is frequently utilized for the evaluation of meningiomas proliferation trend. The expression level of Ki67 demonstrates a positive correlation with the pathological grade, growth rate, peritumoral edema, and recurrence rate of meningiomas. As the size of the meningiomas increases, so does its expression rate; conversely, cases with slower tumor growth and lighter peritumoral edema exhibit lower expression rates. Moreover (36), studies have revealed that patients with recurrent meningiomas exhibit significantly elevated levels of Ki-67 expression compared to those without recurrence, reaching a critical threshold at ~10%.

Numerous evidence demonstrated that most OIM or OSM grow very slowly and are asymptomatic, whereas OIM or OSM occurring in the spinal canal may show clinical symptoms in the early stage due to the narrow space of the spinal canal (2, 37). Compared with ordinary meningiomas, metaplastic meningiomas adhere more heavily to the dura mater or arachnoid membrane, resulting in more difficulty of operation (38, 39).

Tumor resection remains the primary treatment modality for meningiomas (40). However, the surgical management of OIM and OSM is relatively intricate due to extensive tumor adhesion to surrounding brain structures and issues with dural attachment. Additionally, postoperative cerebrospinal fluid leakage and tumor recurrence pose significant challenges to surgical operations, greatly impacting patients' quality of life (40, 41). Therefore, the current recommendation advocates for an individualized approach focusing on achieving maximum and safe resection (40, 42). Studies have indicated that stereotactic radiosurgery (SRS) appears to be a reliable and effective treatment option for recurrent meningiomas and deep-seated lesions where traditional neurosurgical methods are inadequate or ineffective (43, 44). SRS has been clinically applied in various primary and secondary tumors as well as single or multiple meningiomas. With sub-millimeter accuracy, SRS can optimize dose exposure on the target volume compared to conventional radiotherapy techniques while minimizing damage to surrounding critical structures. These characteristics make SRS not only a potential adjunctive therapy but also a valuable alternative in certain cases due to its clinical efficacy and extremely low rate of side effects (45).

The meticulous management of meningiomas is currently under deliberation. The International Consortium on Meningiomas (ICOM) (40) provides several fundamental recommendations. First, although the last decade has witnessed advancements in our understanding of the biology and genomic landscape of meningiomas, further developments are necessary and critical for improving care for patients. Identification of molecular alterations driving the aggressive meningioma phenotype will be critical to advance care for patients and should be done in parallel with the development of reliable preclinical models that allow for rapid translation of discovery to clinical trials. Collaboration with the World Health Organization is needed to advocate for the integration of key molecular alterations that refine standard-of-care classifications to allow for more individualized diagnosis and prognostication such that management and decision-making can be tailored to the patient. In addition to this, standardized core outcomes and definitions that evaluate intervention complication rates, tumor recurrence, seizures, cognitive function, and health-related quality of life are needed to unify language and facilitate the assessment of key metrics in meningiomas. Although most meningiomas requiring treatment will be managed primarily with surgery, particularly challenging cases will likely benefit from review by a multidisciplinary team that can offer the spectrum of various treatment options in meningiomas, including ongoing investigational clinical trials. Lastly, since a subset of patients with meningiomas can have continued impairments that extend beyond the treatment of their tumors, centers of excellence that are able to address the complex needs of these patients in a longitudinal fashion will be key to addressing the unmet needs of this growing population of patients.

However, inspiring, large-scale genomic profiling of meningiomas has uncovered possible driver mutations for a subset of tumors. Several clinical trials are currently underway to evaluate the efficacy of SMO, AKT1, FAK, and mTOR inhibitors in patients with residual, recurrent, or progressive meningiomas (46–48). Furthermore, traditional chemotherapies such as trabectedin are also now being investigated in Phase II trials for use in recurrent higher-grade meningiomas. The increased attention and momentum driving advances in clinical trials in meningiomas are promising and should continue to be a focus of future efforts (40).

In this case, the responsible lesion for clinical symptoms was mainly located in the mass effect of the cervicothoracic spinal cord tumor. Due to its slow growth, the patient presented slowly progressive limb weakness and numbness and was repeatedly diagnosed as “lumbar disease” in other hospitals. A previous study showed that OIM or OSM show are more common in women, and most of the pathological grades are WHO grade 1 (49). Our current study reported a female case pathologically confirmed as OIM and OSM (WHO grade 1), which was consistent with the conclusions of previous studies. The immunohistochemical analysis demonstrated positive expression for EMA, S100, PR, and SSTR2 markers in this patient's sample. Additionally, a low proliferation rate with only 3% Ki67 positivity confirmed the diagnosis as an ossifying meningioma classified as WHO grade 1—indicating its benign nature. Moreover, PR expression predominantly correlated with progesterone levels while maintaining a Ki67 positivity rate below 10%. Notably absent were any signs of significant edema surrounding both intracranial and spinal meningiomas. Considering these imaging and immunohistochemical findings collectively suggests that this patient carries a relatively minimal risk for future recurrence.

The patient was a female with subacute onset, slow progression, and no clinical symptoms at the early stage. With the progression of the disease, the tumor gradually compressed the spinal cord nerves, resulting in paresthesia, limb weakness, and numbness. Histopathological biopsy finally confirmed multiple OIM and OSM (WHO grade 1). According to the latest literature, no more than 50 cases of OIM and OSM have been reported worldwide, and most of them are solitary in the spinal cord (Table 1). It is the first report of multiple OIM and OSM in the spinal cord and cranial cavity. Lastly, it is clear that patients with meningiomas can be affected by both the disease and their treatments, and some have long-lasting effects, resulting in chronic quality-of-life impairments that compound the challenges mentioned above. Consequently, regular brain and spinal cord MRI evaluations have been scheduled annually to closely monitor any potential resurgence.

**Table 1:** Summary of ossified meningioma cases.

**References**	**Age**	**Sex (F/M)**	**Level**	**Tumor number**	**Symptoms**	**Treatment**
Rogers (50)	16	F	T9	1	Myelopathy	Total en bloc tumor resection
Freidberg (51)	69	F	T1-T2	1	Myelopathy	Total en bloc tumor resection
Kandel et al. (52)	17	F	T8	1	Myelopathy	Total en bloc tumor resection
Niijima et al. (23)	75	F	T8-T9	1	Myelopathy	Tumorectomy with dura attachment
Kitagawa et al. (53)	75	F	T9-T10	1	Myelopathy	Total en bloc tumor resection
	60	F	T6-T8	1	Myelopathy	Total en bloc tumor resection
Nakayama et al. (22)	74	F	T9	1	Myelopathy	Total en bloc tumor resection
	45	M	C1-C3	1	Myelopathy	Total en bloc tumor resection
Huang et al. (54)	73	F	T5	1	Myelopathy	Tumorectomy
Saito et al. (55)	54	F	T11	1	Myelopathy	Total en bloc tumor resection
Naderi et al. (37)	15	M	T4	1	Myelopathy	Total en bloc tumor resection
Liu et al. (56)	70	F	T11	1	Myelopathy	En bloc tumor resection
Hirabayashi et al. (57)	82	F	L3	1	Myelopathy	En bloc tumor resection
Tahir et al. (24)	40	F	T6	1	Myelopathy	Total en bloc tumor resection
Uchida et al. (38)	76	F	T8 and T11-T12	2	Myelopathy	En bloc resection with parts of the dura mater and arachnoid
Licci et al. (58)	58	F	T6	1	Myelopathy	Total en bloc tumor resection
Chotai et al. (59)	61	F	T4-T5	1	Myelopathy	Total en bloc tumor resection
Ju et al. (25)	61	F	T9-T10	1	Myelopathy	Total en bloc tumor resection
Taneoka et al. (60)	78	F	T9	1	Myelopathy	Total en bloc tumor resection
Yamane et al. (61)	61	F	T12	1	Myelopathy	Total en bloc tumor resection
Chu et al. (62)	64	F	T9-T10	1	Myelopathy	Total en bloc tumor resection
Demir et al. (21)	26	F	T9-T11	1	Myelopathy	Total en bloc tumor resection
Cochran et al. (63)	47	F	T8	1	Radiculopathy	Total en bloc tumor resection
Xia and Tian (64)	90	M	T10-T11	1	Spinal cord injury after fall	Total en bloc tumor resection
Alafaci et al. (65)	45	M	T2-T3	1	Myelopathy	Gross-total resection of the tumor was achieved in 6 patients while in 3 a subtotal removal of the meningioma was obtained
	75	F	T3-T4	1	Myelopathy	
	86	F	T3-T4	1	Myelopathy	
	65	F	T7	1	Myelopathy	
	72	F	C7	1	Myelopathy	
	40	F	T1-T2	1	Myelopathy	
	65	F	T7-T8	1	Myelopathy	
	40	F	C7	1	Myelopathy	
	41	F	T2-T3	1	Myelopathy	
Prakash et al. (66)	60	F	T7-T8	1	Myelopathy	Total en bloc tumor resection
Sakamoto et al. (67)	57	F	C7	1	Myelopathy	Total en bloc tumor resection
Kim et al. (68)	77	F	T9	1	Back pain, numbness and a progressive	Total en bloc tumor resection
Taneoka et al. (60)	78	F	T9	1	Progressive pain in lower extremities	Tumorectomy with the inner dura
Murakami et al. (26)	29	F	T12	1	Back pain, leg numbness	Total en bloc tumor resection
Taha et al. (2)	22	F	T4-T5	1	Myelopathy	Total en bloc tumor resection
Wang et al. (69)	52	F	T4	1	Back pain	Total en bloc tumor resection
Xu et al. (70)	85	F	T11	1	Back pain, leg pain	Total en bloc tumor resection
Xu et al. (70)	85	F	T11	1	Myelopathy	Total en bloc tumor resection
Buchanan et al. (71)	64	M	T4	1	Myelopathy	Total en bloc tumor resection
Wong et al. (72)	75	F	T10-T11	1	Myelopathy	Total en bloc tumor resection
Thakur et al. (73)	74	F	T8	1	Tingling paresthesia	Total en bloc tumor resection
Dong et al. (3)	76	F	T7-12	5	Myelopathy	Total en bloc tumor resection
Present Case (2023)	34	F	Multiple intracranial and spinal lesions	>10	Weakness and paresthesia in the right leg	Partial tumor resection

## Conclusion

OIM or OSM is a subtype of metaplastic meningiomas that is extremely rare in clinics, and it is more common in women. Most patients with meningiomas grow slowly, and tumors growing in the spinal canal usually have early clinical symptoms. Its clinical symptoms are mainly associated with the location of the tumor, and the imaging manifestations are complex and diverse. The final diagnosis depends on histopathological examination. Due to a few reports of OIM or OSM and most of them are individual cases, there is no large sample of clinical randomized controlled study data. Therefore, the specific mechanism of the occurrence and evolution of OIM and OSM is far from clear.

## Data availability statement

The original contributions presented in the study are included in the article/supplementary material, further inquiries can be directed to the corresponding author.

## Ethics statement

The studies involving human participants were reviewed and approved by the Ethics Committee of the Affiliated Aerospace Hospital of Zunyi Medical University. Written informed consent from the patients/participants or patients/participants' legal guardian/next of kin was not required to participate in this study in accordance with the national legislation and the institutional requirements. Written informed consent was obtained from the individual(s) for the publication of any potentially identifiable images or data included in this article.

## Author contributions

JW, AZ, and BW treated the patient, reviewed the literature, designed the study, and drafted the manuscript. JY, JZ, ML, HL, LC, and PK contributed to the design and implementation of the research. All authors have read, revised, and approved the final manuscript.
